# Moving malaria in pregnancy programs from neglect to priority: experience from Malawi, Senegal, and Zambia

**DOI:** 10.9745/GHSP-D-13-00136

**Published:** 2014-01-27

**Authors:** Elaine Roman, Michelle Wallon, William Brieger, Aimee Dickerson, Barbara Rawlins, Koki Agarwal

**Affiliations:** a Jhpiego, Maternal and Child Health Program (MCHIP), Baltimore, MD, USA; b Jhpiego-Zambia, Lusaka, Zambia; c Johns Hopkins Bloomberg School of Public Health, Department of International Health, Health Systems Program, Baltimore, MD, USA; d Jhpiego, MCHIP, Washington, DC, USA

## Abstract

**Background:**

Pregnant women and infants are particularly vulnerable to malaria. National malaria in pregnancy (MIP) programs in Malawi, Senegal, and Zambia were reviewed to identify promising strategies that have helped these countries achieve relatively high coverage of MIP interventions as well as ongoing challenges that have inhibited further progress.

**Methods:**

We used a systematic case study methodology to assess health system strengths and challenges in the 3 countries, including desk reviews of available reports and literature and key informant interviews with national stakeholders. Data were collected between 2009 and 2011 and analyzed across 8 MIP health systems components: (1) integration of programs and services, (2) policy, (3) commodities, (4) quality assurance, (5) capacity building, (6) community involvement, (7) monitoring and evaluation, and (8) financing. Within each program area, we ranked degree of scale up across 4 stages and synthesized the findings in a MIP table of analysis to reveal common themes related to better practices, remaining bottlenecks, and opportunities to accelerate MIP coverage, strengthen MIP programs, and improve results.

**Findings:**

Each of the 3 countries has malaria policies in place that reflect current MIP guidance from the World Health Organization. The 3 countries successfully integrated MIP interventions into a platform of antenatal care services, but coordination at the national level was disjointed. All 3 countries recognized the importance of having a MIP focal person to ensure collaboration and planning at the national level, but only Malawi had appointed one. Commodity stockouts were frequent due to problems at all levels of the logistics system, from quantification to distribution. Lack of support for quality assurance and weak monitoring and evaluation mechanisms across all 3 countries affected optimal coverage.

**Conclusions:**

MIP programs should address all 8 interconnected MIP health systems areas holistically, in the context of a health systems approach to building successful programs. The MIP table of analysis can be a useful tool for other malaria-endemic countries to review their programs and improve MIP outcomes.

## Abstract (precis)

Program areas that were generally working well in malaria in pregnancy programs (MIP) included: (1) integration of MIP interventions into antenatal care; (2) development of up-to-date policies; (3) active involvement of communities; and (4) development of capacity-building materials for training. Challenges remain in the areas of: (1) commodities; (2) quality assurance; (3) monitoring and evaluation; and (4) financing.

## BACKGROUND

Pregnant women are particularly vulnerable to malaria infection, which has a “trickle-down” negative effect from the mother to the fetus and newborn. Malaria in pregnancy (MIP) contributes to maternal anemia, maternal death, stillbirth, spontaneous abortion, and low birth weight.^1^^–^^3^ In areas of stable malaria transmission, babies are more likely to be small for gestational age, and in areas of unstable malaria transmission, they are more likely to be born preterm. One-third of all newborn deaths and an estimated 11% of neonatal deaths in malaria-endemic regions of Africa are due to low birth weight associated with *Plasmodium falciparum* infections during pregnancy.^4^ Approximately 125 million pregnancies occur each year in areas with *P. falciparum* and/or *Plasmodium vivax* malaria transmission; 10,000 of these women and 200,000 of their newborns will die as a result of MIP.^5^^,^^6^

Malaria in pregnancy contributes to both maternal and infant mortality and morbidity.

In 2004, the World Health Organization (WHO) *Regional Office for Africa* developed a framework to prevent and control malaria during pregnancy. The framework promotes a 3-pronged approach in stable malaria transmission areas^2^:

1. Intermittent preventive treatment of pregnant women (IPTp) with the antimalarial drug sulfadoxine-pyrimethamine (SP)
2. Use of insecticide-treated bed nets (ITNs)
3. Effective case management among pregnant women showing signs and symptoms of malaria

Reflecting the latest evidence that frequent dosing of IPTp-SP is effective in reducing the consequences of MIP,^7^ WHO updated its guidance in October 2012 to recommend IPTp with SP in areas of moderate to high malaria transmission for all pregnant women at each scheduled antenatal care (ANC) visit.^8^ (WHO recommends at least 4 ANC visits.) WHO also recommends:

- The first IPTp-SP dose should be administered as early as possible during the second trimester of gestation.
- Each SP dose should be given at least 1 month apart.
- The last dose of IPTp-SP can be administered up to the time of delivery without safety concerns.

At the 2000 African Summit on Malaria held in Abuja, Nigeria, 44 African nations committed to the goal that 60% of pregnant women at risk of malaria would be covered with the above MIP interventions by 2005.^9^ Since 2005, global targets for IPTp have been set even higher at 85% and 100% by the President's Malaria Initiative (PMI) and the Roll Back Malaria Initiative, respectively.^10^^,^^11^

The global health community has committed to achieving at least 85% coverage of intermittent preventive treatment of pregnant women with antimalarial drugs.

In the last decade, 39 African countries,^12^ in which malaria transmission ranges from moderate to high and the dominant malaria species threat is *P. falciparum*, have revised national policies and supported MIP programs in line with WHO's 3-pronged approach. However, many countries are far from achieving their targets for both IPTp uptake and ITN use. Van Eijk et al. found 45 of 47 countries surveyed (96%) had a policy for distribution of ITNs for pregnant women; 39 of 47 countries (83%) had an IPTp policy; in 2007, an estimated 6.4 million of 25.6 million pregnant women (25%) received at least 1 treatment dose and 19.8 million (77%) visited an antenatal clinic (31 countries). Estimated coverage was lowest in areas of high-intensity transmission of malaria.^12^

Hill et al. systematically examined both demand and supply factors associated with low coverage of IPTp uptake and ITN use. In some countries, they found poorer women, women with no education, and women living in rural areas were significantly less likely to have IPTp coverage or an ITN. Also, not surprisingly, women's pattern of ANC use was a more important determinant of IPTp uptake than for ITN ownership. ANC use was affected by provider performance, organizational problems at the facility, health system weaknesses beyond ANC, and factors that cannot be controlled by the health system, such as pregnant women delaying ANC attendance.^13^

In light of most countries' suboptimal progress, we conducted MIP case studies in Malawi (2011),^14^ Senegal (2011),^15^ and Zambia (2010)^16^ to learn more about what is needed to enable health systems to meet MIP targets. (Malawi adopted an IPTp policy in 1993 and an ITN policy for pregnant women in 2002. Senegal and Zambia adopted IPTp policies in 2001 and ITN policies for pregnant women in 2000.^12^)

In addition to being PMI focus countries, these 3 countries were selected because, although they have not achieved target MIP goals, they have been more successful than other sub-Saharan African nations in increasing IPTp uptake and, to some extent, ITN use. Using a positive deviance approach,^17^ the purpose of the case studies was to assess how these 3 countries were able to achieve relatively greater progress in MIP control, as well as to understand how the countries ranked in terms of readiness with respect to 8 key MIP health systems areas that are building blocks within any MIP program (Box).

**BOX. Malaria in Pregnancy Program Areas**

**INTEGRATION**

MIP is a core component of focused antenatal care (FANC) services.^18^^,^^19^ For MIP programs, integrated services include collaboration between national reproductive health programs and national malaria control programs as well as other national programs delivering care through comprehensive reproductive, maternal, and child health services—such as national HIV/AIDS and tuberculosis programs—to: (1) ensure harmonized policies, guidelines, and training material; and (2) coordinate effective program implementation.

**POLICY**

Country MIP policies that are based on the latest scientific evidence and include defined goals as well as national guidelines to achieve those goals set the stage for effective MIP implementation. Policies need to be harmonized and effectively integrated across different divisions of the health sector as described under “integration.”

**COMMODITIES**

Commodities include not only correct medicines and medical products but also having systems in place to ensure availability of commodities at the point of service. For MIP, this includes availability of SP, cups and water for IPTp, and long-lasting ITNs at ANC. It also includes diagnostic tools, rapid diagnostic tests and/or microscopy, and treatment medicines to effectively provide case management to pregnant women with signs of malaria.

**QUALITY ASSURANCE**

Quality assurance is a wide-ranging concept covering all matters that individually or collectively influence the efficacy, safety, appropriateness, and acceptability of services. For MIP, this includes performance standards to measure quality of care, supervision support to improve the quality of care, and self-assessment by health care providers to monitor their own delivery of services.

**CAPACITY BUILDING**

Capacity building is defined as strengthening human resources by improving knowledge and skills. For MIP, this includes competency-based preservice and in-service training that reflects current WHO guidance and national policies.^20^

**COMMUNITY AWARENESS AND INVOLVEMENT**

Community engagement is defined as the involvement of communities in the promotion and/or delivery of health services. For MIP, this includes raising awareness about MIP prevention and control; for example, early attendance at ANC, promotion of IPTp and ITNs, and effective case management. Availability of resources and effective monitoring of health promotion are key components of this program element.^21^

**MONITORING AND EVALUATION**

Monitoring and evaluation, when effective, will capture MIP service delivery indicators correctly and will, on a regular basis, feed into a national health management information system. These data will be used for decision-making at all points of care and at the national level for policy formulation. National-level surveys to evaluate the coverage and health impact of MIP programs are a critical component of programs to help understand trends in health outcomes and drive program decision-making.^22^

**FINANCING**

Financing includes a combination of national government and donor funding that guarantees MIP programs receive the support and resources they need to consistently reach all pregnant women.

The specific case study objectives were to identify:

1. Promising practices/strategies that have supported MIP programming success
2. Existing bottlenecks in MIP program implementation and how these are addressed
3. Lessons learned that can inform future MIP programming in other countries

## METHODS

### Study Design

We used a systematic case study research methodology to analyze the factors contributing to successes in national MIP programs in Malawi, Senegal, and Zambia, based on the 8 health system areas, as well as challenges that have hindered further progress. This type of research is well-suited for exploring complex issues in depth, such as health systems functioning, and can help illuminate findings from previous quantitative research, such as household and facility surveys.^23^ In addition, this approach allowed us to draw general conclusions while comparing different countries.

### Data Collection

Data collection methods in each country consisted of a comprehensive desk review of the literature and secondary data, followed by qualitative key informant interviews with national MIP stakeholders, including representatives from the National Malaria Control Programme (NMCP), National Reproductive Health Programme (NRHP), staff from the U.S. Agency for International Development (USAID) and PMI, and implementing organizations supporting the Ministry of Health. Key informants were selected using both purposive and snowball sampling techniques.

For the desk review, we identified such documents as national policies and guidelines—for both malaria and reproductive health, where comprehensive ANC services, including MIP, are provided to pregnant women—as well as national progress reports and assessments. We also reviewed applications and progress reports for the Global Fund to Fight AIDS, Tuberculosis and Malaria (Global Fund), PMI Malaria Operational Plans, peer-reviewed articles, implementing partner reports, national health management information system (HMIS) reports and forms, as well as Demographic and Health Surveys and Malaria Indicator Surveys.

Information from the initial desk review was used to identify gaps in program-related MIP information and areas requiring further elucidation. This informed the development of qualitative key informant interview guides with input from stakeholders from the NMCP and the NRHP. A set of questions, linking back to the 8 health system areas of MIP, was developed for the first country (Zambia). The questions were not pilot tested but were refined for use in subsequent countries, adapting them to the country context and to the specific stakeholder's area of MIP support and job function. For example, key informants from organizations supporting logistics management were primarily asked questions related to procurement and distribution of MIP commodities.

Data were collected in Zambia from September 2009 to October 2009; in Senegal, in March 2010; and in Malawi, in January 2011. The length of the interviews ranged from approximately 60 minutes to 90 minutes, and information from respondents was recorded on paper questionnaires and then transcribed on the same day into a Microsoft Word document. When information was unclear or information from stakeholders was conflicting, the interviewees were given the opportunity to review the draft report and provide clarification.

### Data Analysis

The study team conducted content analyses for the secondary and qualitative data for each country using the 8 MIP health system areas. Within each MIP program area, the study team determined degree of scale up by ranking progress made along 4 stages (Figure). A rating of 1 means nothing to little had been done to support the health system area; 2 suggests some efforts supported the health system area; 3 means the country was supporting the health system area and some progress had been achieved; and 4 means the health system area had been fully scaled up. Generally, higher scores are identified with better practices and lower scores help to identify remaining challenges. This program rank, or “program effort score,” is derived from similar methodologies used in the fields of family planning and maternal health, which link program effort with outcome indicators, such as contraceptive prevalence and total fertility rate.^24^^,^^25^ In the case of the MIP case studies, the program rank is linked with IPTp uptake and ITN use.

Degree of scale up within each MIP program area was ranked on a scale of 1 (nothing to little had been done) to 4 (full scale up).

**FIGURE.**
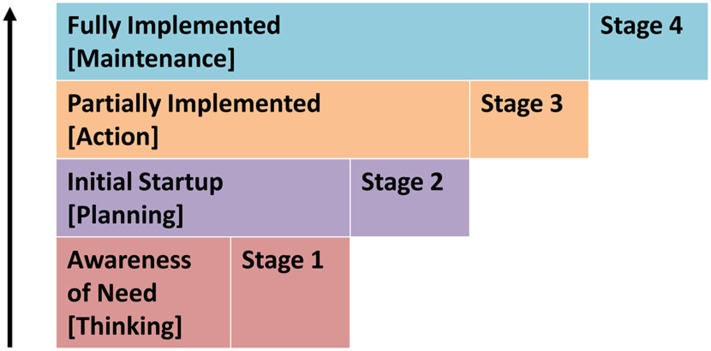
Stages of Malaria in Pregnancy Program Implementation

Few differences in opinion among the study team were encountered in terms of how each health system area should be rated; however, when they did occur, discussions among researchers and authors of all 3 country case studies were held, using the MIP matrix as the guide to rate all countries and elements fairly. Information was coded and organized manually, and anonymous quotations were selected to illustrate key points while preserving participant privacy.

In this article, we synthesized the findings across the 3 MIP case studies conducted in Malawi,^14^ Senegal,^15^ and Zambia^16^ to draw together common themes related to: (1) better practices, (2) remaining bottlenecks, and (3) opportunities to accelerate coverage in MIP programming.^19^^–^^21^ This led to the development of an “Expanded MIP Table of Analysis” (Table 1), directly building on the “Stages of MIP Program Implementation Matrix.”^26^ The “Expanded MIP Table of Analysis” includes key indicators for each health system area and objective ratings that allowed 5 reviewers to review the evidence in detail.

**TABLE 1.** Expanded MIP Table of Analysis

**Indicator**	**Stage 1: Awareness of Need [Thinking]**	**Stage 2: Initial Startup [Planning]**	**Stage 3: Partially Implemented [Action]**	**Stage 4: Fully Implemented [Maintenance]**
**Integration**				
Central-level MIP programming is harmonized among RH, malaria control, and HIV programs.	National stakeholders in RH, malaria control, and HIV are aware of the need to harmonize their materials.	National stakeholders in RH, malaria control, and HIV have discussed planning to harmonize their materials.	National stakeholders in RH, malaria control, and HIV have started to harmonize their materials.	National RH, malaria, and HIV materials have been harmonized.

Central-level MIP programming is coordinated among RH, malaria control, and HIV programs.	National stakeholders in RH, malaria control, and HIV are aware of the need to coordinate program implementation.	National stakeholders in RH, malaria control, and HIV meet on a regular basis to share program strategies.	National stakeholders in RH, malaria control, and HIV meet on a regular basis to coordinate program implementation.	National stakeholders in RH, malaria control, and HIV leverage resources across program areas and implement activities jointly.

MIP services are integrated with FANC, PMTCT, and malaria control services at the facility level.	National stakeholders and relevant health care providers are aware of need to integrate MIP with FANC, PMTCT, and/or malaria control services.	MIP guidelines are standardized and integrated across FANC, PMTCT, and malaria control service delivery guidelines and preservice and in-service curricula.	Relevant health care providers have been trained in and are integrating MIP services with FANC and PMTCT but are constrained by commodity stockouts.	Relevant health care providers are trained in and are integrating MIP services with FANC and PMTCT.

**Policy**				
National policy on MIP exists.	Relevant stakeholders in MCH/RH, HIV, and malaria are aware of need for MIP policy.	MIP policy has been drafted and includes WHO's 3-pronged approach, as appropriate, with involvement and acceptance of relevant stakeholders.	MIP policy has been disseminated to major stakeholder organizations.	All service units (public, nongovernmental, and private) are providing the components of care and services based on national MIP policy.

National guidelines and/or performance standards have been developed.	Relevant stakeholders are aware of need for guidelines and standards for MIP.	MIP guidelines and standards have been drafted and include WHO's 3-pronged approach, as appropriate, with involvement and acceptance of relevant stakeholders.	MIP guidelines and standards have been disseminated to major stakeholder organizations and health care providers.	All service units (public, nongovernmental, and private) are providing care and services in accordance with national MIP guidelines and standards.

National strategy and action plans for malaria and RH include MIP programming.	Relevant stakeholders are aware that strategies and action plans for malaria and RH need to include MIP.	MIP programming has been integrated into strategies and action plans that include WHO's 3-pronged approach, as appropriate, with involvement and acceptance of relevant stakeholders.	MIP strategies and action plan components have been disseminated to major stakeholder organizations.	All service units (public, nongovernmental, and private) are implementing MIP activities in accordance and on schedule with strategies and action plans.

**Commodities**				
The health system at all levels is procuring WHO- recommended commodities for prevention and case management of MIP.	MIP commodities and supplies appear in broad essential medicines lists but are not congruent with current WHO guidance.	An updated essential drug list includes only those MIP commodities approved by WHO.	Antenatal and primary health care clinics stock WHO-recommended MIP commodities, but some unapproved medicines remain on shelves.	Only WHO-approved MIP medicines and supplies are stocked in antenatal and primary health care clinics.

Systems are in place to guarantee regular provision of MIP commodities to ANC clinics as part of routine service delivery.	Current procurement and supply system does not guarantee MIP commodities, but stakeholders are discussing problems in the system.	Improved supply chain management systems are being designed, including training on procurement for relevant staff at all levels.	Approved MIP medicines and supplies are being provided to ANC and primary health care clinics, but stockouts occur.	Approved MIP medicines and supplies are available on a regular basis at ANC and primary health care clinics.

**Quality Assurance**				
Supervisory and performance assessment tools, including performance standards, are developed for MIP programming.	Relevant stakeholders are aware of the need for MIP supervisory and performance assessment tools that are based on MIP policies and guidelines.	Supervisory and performance assessment tools have been developed and harmonized in line with national MIP policies and guidelines.	Supervisory and performance assessment tools have been disseminated to all supervisors, in-charges, and health care providers.	Supervisory and performance assessment tools are consistently used in routine performance assessments.

Regular supervisory visits and quality assessments are being conducted.	Relevant stakeholders are aware of the need to conduct regular MIP supervisory visits and quality assessments for relevant health care providers, using tools based on MIP policies and guidelines.	Supervisory and performance assessment tools have been developed and harmonized in line with national MIP policies and guidelines.	Supervisors and trainers have been trained in supervisory and performance assessment tools and are supervising health care providers on a limited basis.	Supervisors are routinely providing supervision to health care providers based on national supervisory and performance assessment tools.

Self-assessments are being conducted.	Relevant stakeholders are aware of the need for ongoing self-assessment by relevant health care providers using MIP supervisory and performance assessment tools based on MIP policies and guidelines.	Supervisory and performance assessment tools have been developed and harmonized in line with national MIP policies and guidelines.	Relevant health care providers are trained in and are performing self-assessments using performance standards on a limited basis.	Relevant health care providers are trained in and are using self-assessment tools routinely.

**Capacity Building**				
IST on MIP is organized and provided.	Relevant stakeholders are aware of the need to adapt MIP policies and guidelines into IST for relevant health care providers.	MIP IST curriculum has been developed in line with national policies and guidelines with involvement and acceptance of relevant stakeholders.	Training is in initial phases of rollout with national plans for scale up.	IST training in MIP, in line with national policy and guidelines, is currently ongoing for relevant staff in all service units (public, nongovernmental, and private), including through integration with FANC, malaria control, and/or PMTCT.

PST on MIP is organized and provided.	Relevant stakeholders are aware of the need to adapt MIP policies and guidelines into PST for relevant service providers.	MIP PST curricula has been developed in line with national policies and guidelines with involvement and acceptance of relevant stakeholders.	Training of trainer teams from among the relevant stakeholders has been undertaken, and instructors, tutors, and preceptors have been trained.	Students in all training institutions (public, nongovernmental, and private) have received MIP PST in line with national policies and guidelines.

**Community-Based MIP Programs**				
Interventions are in place to promote community awareness, education, and communication about MIP and its control options.	No community awareness activities have been planned, but partners have been consulted about designing these.	MIP communication plan and guidelines have been developed, and materials are being designed and channels selected.	MIP communication activities are being aired and disseminated through mass media and community-based interpersonal communication.	MIP awareness and communication activities are sustained through the media, health centers, community volunteers, and other channels.

Resources are provided so that the community itself can take action to control MIP.	Policies are being debated to enable community involvement in MIP prevention and case management.	Community MIP prevention and case management guidelines are adopted; training and supply processes are being set up to ensure communities have the skills and resources needed for action in MIP prevention and case management.	Community members have been trained on MIP prevention and case management and provided initial resources and supplies to undertake MIP prevention and case management activities.	Wide coverage of MIP prevention and case management resources are sustained in the communities to guarantee universal access to MIP prevention and case management.

Data are collected and disseminated that show the community has increased uptake of MIP program interventions.	Partners are discussing ways to monitor the results of community awareness and action in MIP programming.	Specific data collection tools have been designed and pretested for documenting uptake of MIP interventions resulting from community action, including a process by which such data are incorporated into facility, district, and national HMIS.	Malaria data collection forms have been distributed to communities and community members have been trained on use of the forms.	Communities are submitting malaria forms regularly to nearest health facility where information is summarized and submitted to HMIS as well as being used to make decisions to improve community MIP interventions.

**Monitoring and Evaluation**				
MIP control data are collected routinely and used for decision making at all points of care (facility, district, regional).	Partners are discussing ways to monitor the results of MIP care and action in MIP programming.	Data collection tools have been designed and pretested for documenting uptake of MIP interventions delivered at health facilities.	Malaria data collection forms have been distributed to health facilities, and health care providers have been trained on use of the forms and are using them routinely.	Health facilities are reporting MIP data regularly to the district level where the information is summarized and passed on to the national level. MIP information is being used to make decisions to improve MIP interventions and care at national and lower levels.

National population-based surveys are collecting data on trends in MIP intervention coverage and health outcomes.	National governments are aware of the need to prioritize MIP into national level surveys.	National governments and partners are committed to the inclusion of WHO-promoted MIP indicators into national surveys.	National surveys include WHO-promoted MIP indicators.	National surveys are being used to discern trends in MIP intervention coverage and health outcomes, and data are used for policy dialogue and planning.

**Financing**				
National governments are providing funding to MIP programs.	National governments are aware of the need to prioritize MIP in their annual program funding.	National governments commit funding that is not yet adequate to support projected costs.	National governments contribute significant funding that supports majority of projected costs for MIP priorities in their national malaria strategies.	National governments have committed and disbursed funds to MIP programs that are adequate for all projected costs.

Donors are investing in national MIP programs.	Donors are aware of the need to prioritize funding for MIP programs.	Governments are submitting proposals for funding and limited donor funding exists.	Governments are successful in soliciting support and strong donor funding exists.	Ample donor funding exists for MIP and is being used effectively.

Abbreviations: ANC, antenatal care; FANC, focused antenatal care; IST, in-service training; HMIS, health management information system; MCH, maternal and child health; MIP, malaria in pregnancy; PMTCT, prevention of mother-to-child transmission of HIV; PST, preservice training; RH, reproductive health; WHO, World Health Organization.

## FINDINGS

A total of 43 key informants were interviewed across the 3 countries (15 in Malawi, 18 in Senegal, and 10 in Zambia), representing a diverse group of national stakeholders in each country involved in MIP service delivery. No one who was asked to participate refused, and no one ended their interview early.

Table 2 highlights key outcomes for MIP—uptake for IPTp uptake, ITN use, and ANC attendance—in Malawi, Senegal, and Zambia. ANC attendance (for at least 1 visit) was over 90% in all 3 countries, and at least half of pregnant women received 2 or more doses of IPTp for malaria prevention. ITN use among pregnant women, however, was much lower, ranging from 29% in Senegal to 46% in Zambia.

**TABLE 2.** Key MIP Outcomes: National IPTp Uptake, ITN Use, and ANC Attendance

**Indicator**	**Malawi**	**Senegal**	**Zambia**
Pregnant women receiving 2 or more doses of IPTp for malaria prevention, %	60.3^a^	52.2^c^	70.2^a^
Pregnant women sleeping under an ITN, %	35.3^a^	28.5^c^	45.9^a^
Households with at least 1 ITN, %	56.8^a^	63.3^c^	64.3^a^
Pregnant women attending at least 1 ANC visit, %	96.5^b^	91.1^d^	93.7^e^
Pregnant women attending more than 1 ANC visit, %	94.9^b^	87.3^d^	94.3^e^

Abbreviations: ANC, antenatal care; IPTp, intermittent preventive treatment of pregnant women; ITN, insecticide-treated bed net; MIP, malaria in pregnancy.

a 2010 Malaria Indicator Survey.

b 2010 Demographic and Health Survey.

c 2008/9 Malaria Indicator Survey.

d 2005 Demographic and Health Survey.

e 2007 Demographic and Health Survey.

Table 3 shows each country's stage of implementation by MIP health system area, based on the scale of 1 to 4 described earlier. Out of a perfect score of 4, the overall average score was 2.8 for Malawi, 3.1 for Senegal, and 2.9 for Zambia. Issues with **commodities**, **quality assurance**, and **financing** accounted for the most significant challenges across countries, with scores generally ranging from 2.0 to 2.5 in these program areas. Each program area is described in more detail below.

Problems with commodity stockouts, quality assurance, and financing accounted for the most significant MIP program challenges in the 3 countries.

**TABLE 3.** National Stage of MIP Program Implementation by Health System Area, Malawi, Senegal, Zambia, 2010–2011

**Country**	**MIP Health System Area**							
	**Integration**	**Policy**	**Commodities**	**Quality Assurance**	**Capacity Building**	**Community Awareness & Involvement**	**Monitoring & Evaluation**	**Financing**
**Malawi**	Weak collaborationamong MOH, RHU, and NMCP, resulting in disjointedand duplicative MIP programming	MIP policies in line with WHO guidelines but discrepancies across national documents in administration of IPTp	Frequent stockouts of SP and ITNs at ANC clinics hampering uptake of interventions	Limited diagnostic capacity, mistrust of SP efficacy, and irrational use of SP, leading to inconsistent application of clinical guidelines	Capacity-building efforts with current MOH/NMCP and health facility personnel have limited impact in situations of chronic understaffing	Late initiation of ANC (after first trimester), limiting number of IPTp doses administered to pregnant women	Weak HMIS and low provider investment in data management, leading to poor data quality	Government has committed some funds to MIP programs but still relies heavily on donor support
**Score**	**2.5**	**3.0**	**2.5**	**2.5**	**3.5**	**3.0**	**3.0**	**2.5**

**Senegal**	Joint program planning among NRHP, NMCP, and NACP is low, resulting in duplication of program efforts	MIP policies in line with WHO guidelines, with widespread dissemination to providers	Frequent stockouts of SP and ITNs at ANC clinics hampering uptake of interventions	MIP clinical performance standards developed but low level of supervision due to lack of human resources and logistical and financial constraints	MIP content up-to-date in preservice and in-service educational materials but redundancies in training among NRHP, NMCP, and NACP	Community groups are engaged in promotion of use of ITNs and IPTp; late initiation of ANC (after first trimester), limiting number of IPTp doses administered to pregnant women	Improved data quality through a web-based HMIS; 2 WHO-recommended MIP indicators not tracked	Government has committed some funds to MIP programs, fully funding SP, but still relies heavily on donor support, especially for ITNs
**Score**	**3.0**	**4.0**	**2.0**	**2.0**	**3.0**	**3.5**	**4**	**3**

**Zambia**	Weak linkages among MOH, RHU, NMCP, and MOH PMTCT Unit limit leveraging of funds and development of holistic MIP package	MIP policies updated in line with WHO guidelines and consistently integrated across national documents	Frequent stockouts of SP and ITNs at ANC clinics hampering uptake of interventions; lack of hemocues limiting hemoglobin testing	Routine, quality supportive supervision for service providers needed to ensure adherence to MIP guidelines	Capacity-building efforts with current MOH/NMCP and health facility personnel have limited impact in situations of chronic understaffing	Late initiation of ANC (after first trimester) limiting number of IPTp doses administered to pregnant women	Inconsistent and inaccurate recording of facility-level data by service delivery providers, leading to poor data quality	Government has committed some funds to MIP programs but still relies heavily on donor support
**Score**	**3.0**	**4.0**	**2.0**	**2.0**	**4.0**	**3.0**	**3.0**	**2.0**

Abbreviations: ANC, antenatal care; HMIS, health management information system; IPTp, intermittent preventive treatment of pregnant women; ITN, insecticide-treated bed net; MIP, malaria in pregnancy; MOH, Ministry of Health; NACP, National AIDS Control Programme; NMCP, National Malaria Control Programme; NRHP, National Reproductive Health Programme; PMTCT, prevention of mother-to-child transmission of HIV; RHU, Reproductive Health Unit; SP, sulfadoxine-pyrimethamine; WHO, World Health Organization.

### Integration of Programs and Services

After the 2000 Abuja Summit, all 3 countries successfully brought together malaria control and reproductive health policy makers and program implementers, through malaria or MIP-specific working groups, to institute MIP policies and interventions. In line with WHO recommendations, the countries successfully integrated MIP interventions into a platform of ANC services at the facility level—a key step in reaching the current levels of coverage.

Since this initial rollout, however, political will has waned or been diverted elsewhere, and while services at the facility level remain integrated, national-level coordination and planning has become disjointed. At the time of data collection, MIP working groups in Malawi and Zambia were reported to be dissolved or inactive, not due to a conscious decision of stakeholders but due to competing health priorities. The Malawi NMCP did, however, have a MIP focal point person on staff who reportedly intended to revitalize the link between the NMCP and the Reproductive Health Unit (RHU). Zambia had been seeking funding for a MIP focal point person but, at the time of this writing, had yet to succeed. In Zambia, a lack of human resources in the Ministry of Health was reported to be a major constraint in cross-sector coordination. The RHU and the NMCP confirmed that although representatives of the other unit are invited to participate in joint annual planning, mutual participation in the planning process has been minimal for the last several years because there are not enough staff to attend each other's meetings. This reportedly resulted in the shifting of MIP funds to other priority areas of malaria implementation.

In Senegal, one interviewee noted:

… national-level integration may be less effective because coordination of national activities is not routine … and the level of joint planning [between the NRHP and NMCP] is low.

This lack of coordination negatively affects program components, including training capacity, supervision, and availability of commodities. In Malawi and Senegal, lack of coordination has resulted in parallel, duplicative MIP trainings, conducted independently by different groups for the same health care providers. At the time of this article, a focused antenatal care (FANC) working group had been created in Zambia to address issues in MIP programming; however, the group has struggled to accomplish tasks because the RHU, NMCP, and implementing partners have limited funding and staff to support MIP and FANC.

**Figure:**
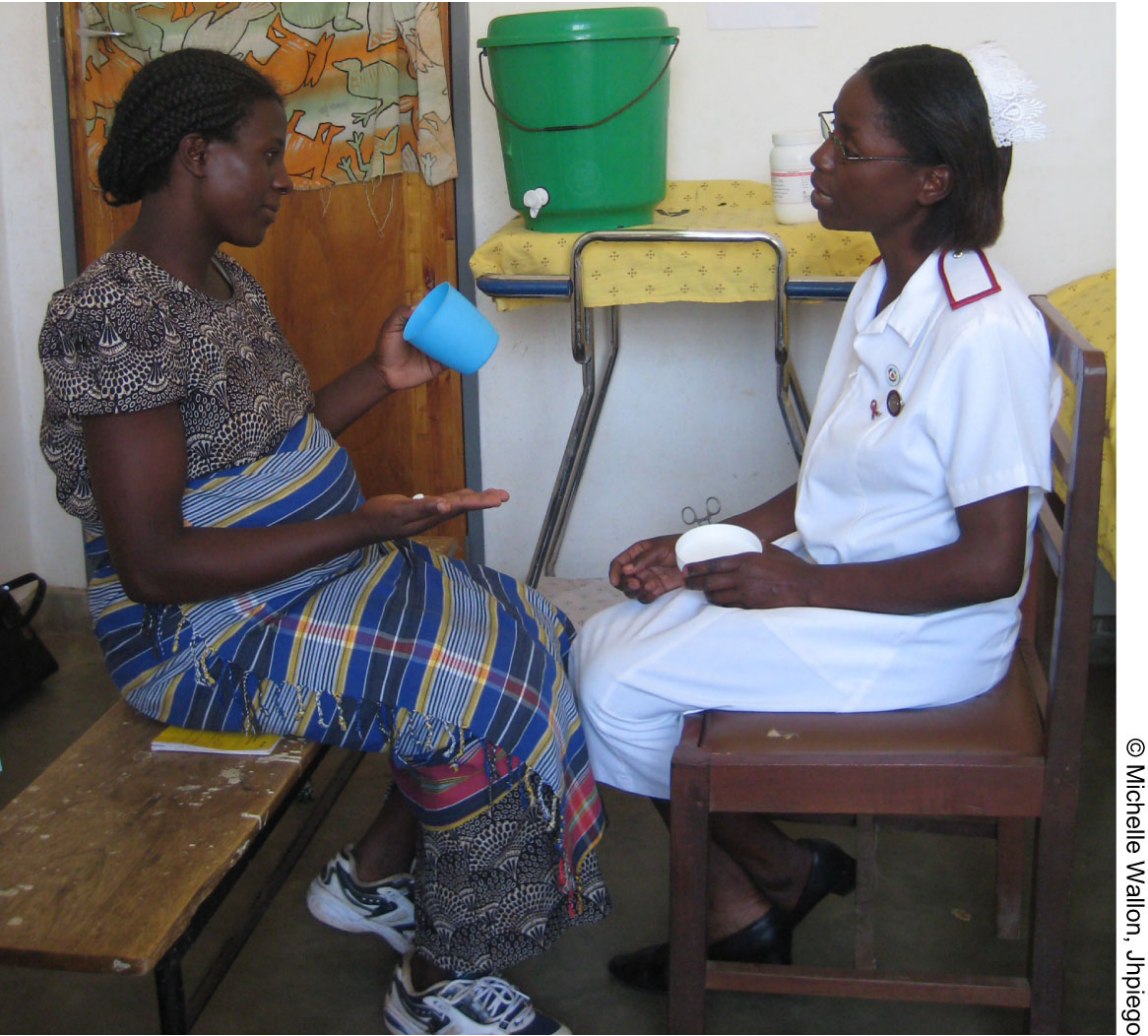
In Malawi, a pregnant woman receives intermittent preventive treatment for malaria at an antenatal care visit.

### Policy Processes

All 3 countries have malaria policies in place that reflect WHO MIP guidance. In Senegal and Zambia, national reproductive health and malaria control policies and guidelines have been harmonized through strong leadership by the NMCPs and commitment from partners to support the process. Dissemination of national guidelines in both Senegal and Zambia has been widespread. In Zambia, specifically, the NMCP conducted case management updates to reorient providers, addressing issues such as the correct use of SP, and, at the time of data collection, planned to distribute revised treatment guidelines with a memorandum to district-level staff for dissemination to providers.

In Malawi, there are inconsistencies between reproductive health and malaria guidelines. For example, the national reproductive health service delivery guidelines stipulate IPTp provision at defined week intervals, while the national malaria guidelines stipulate IPTp provision following quickening and giving at least 3 doses 1 month apart. The Malawi reproductive health guidelines also stipulate that providers should not administer SP after 36 weeks gestation, while the malaria guidelines note no such limitation. Providers may provide different care depending on which set of national guidelines they refer to. What providers actually practice in all 3 countries is unknown as documentation of findings during supervision visits was not available.

### Commodities

**Sulfadoxine-pyrimethamine:** In each of the 3 countries, WHO-recommended medicines for malaria treatment for pregnant women and for IPTp are approved and available through ANC clinics. However, stockouts of SP at ANC clinics were frequent due to central-level stockouts and ineffective distribution systems. This problem was further exacerbated by the irrational use of SP (based on key informant interviews) to treat malaria cases in the general population, whereas SP should be reserved for preventing malaria in pregnant women. In Zambia, although NMCP partners routinely provided the Ministry of Health with early warnings of impending stockouts of SP, stockouts persisted. This happened in 2009, when one implementing partner had to shift US$50,000 meant for rapid diagnostic tests to procure an emergency stock of SP.

Stockouts of antimalaria drugs and bed nets were frequent.

One stakeholder interviewed in Zambia described the situation:

Sometimes you go to a clinic and they are stocked out of SP and they say it is because there isn't any at the district. Then, you go to the district and find it. Sometimes, they are stocked out at the district, but you find it at the central level.

In Malawi and Senegal, stakeholders described similar issues with persistent SP stockouts. Stakeholders cited problems at all levels of the logistics management system for commodities, including quantification, ordering of drugs in a timely manner, tendering, receipt, storage, and distribution.

**Insecticide-treated bed nets:** In the 3 countries, distribution of nets to pregnant women free of charge at ANC clinics is considered an effective mechanism for increasing usage among pregnant women and for incentivizing ANC attendance. Nevertheless, as with SP, facilities often experienced ITN stockouts due to procurement shortfalls, central-level stockouts, and disjointed distribution systems.

In Malawi, it was reported that ITNs were frequently, but not always, available in ANC clinics. Facility supplies were based on estimated target populations rather than consumption, but recent changes from a single- to a multi-distributor system led to increased stockouts. At the time of data collection, all 3 countries were planning to implement universal ITN coverage campaigns, aimed at increasing net usage by the general population, and supplemental efforts targeting pregnant women and children under 5 years of age.

**Figure:**
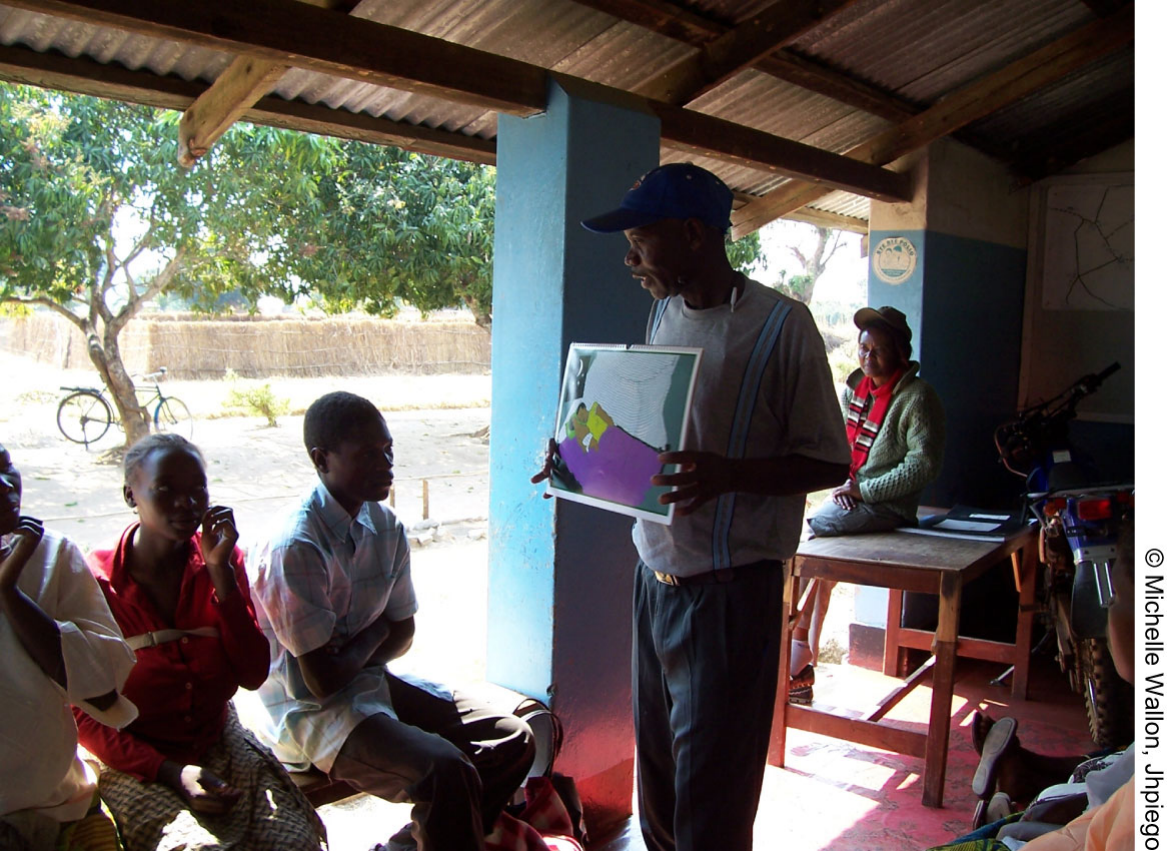
In Zambia, a counselor educates community members at an antenatal care clinic about use of insecticide-treated bed nets.

### Quality Assurance

Due to lack of funding and competing responsibilities among Ministry of Health staff, comprehensive quality assurance systems were not currently functioning in any of the 3 countries. While MIP clinical performance standards had been developed in each of the countries, application of these standards was inconsistent. In large part, each country relies on routine supervision from national, provincial, or district-level supervisors; however, due to lack of human resources and logistical and financial constraints, these visits were not consistently conducted. In 2006, Zambia developed an integrated reproductive health tool composed of reproductive health standards that included MIP (for example, group and individual education on MIP, ITN use, and IPTp uptake). Provincial-level Ministry of Health staff were oriented to this tool with the intention that training would cascade down to district- and facility-level staff. At the time of data collection, it was unknown if district-level orientations had been completed and, according to stakeholder reports, the tool was not in widespread use.

Quality assurance systems for MIP programs were not functioning in any of the 3 countries.

Although Malawi has designed an integrated tool for quarterly supervision visits that targets infection prevention, MIP, reproductive health, and prevention of mother-to-child transmission of HIV, these visits were occurring infrequently. In Malawi, one interviewee mentioned:

*Donors place more emphasis on “number of persons trained” in program-reporting indicators than on the number supervised or the impact of training those persons, with program design following suit*.

In Senegal, the program review revealed that 31% of health facilities did not receive any kind of supervision.

Lack of supervision has negative implications for consistent delivery of quality services for pregnant women as well as the general population. This can contribute to SP stockouts as well as to increased morbidity and mortality from improperly treated non-malarial fevers. For example, in Malawi and Zambia, stakeholders reported that SP was often used by providers to treat fevers in men, children, and non-pregnant women; in Zambia, SP was given when rapid diagnostic tests were negative; and in Malawi, SP was given when there were stockouts of artemisinin-based combination therapies (ACTs).

### Capacity Building

Malawi, Senegal, and Zambia have all updated in-service training and preservice education materials with MIP content. This positions each country to focus training on evidence-based updates and maintenance of critical MIP competencies. However, as noted previously under the integration findings, at the time of data collection, there was a need to harmonize efforts further to avoid redundancies in training, both between preservice education and in-service training and within in-service training courses.

Lack of coordination at the national level contributed to redundancies in training.

In Malawi, the RHU and NMCP were implementing parallel in-service training events. As one interviewee pointed out:

Where is the rocket science in MIP? We present focused ANC like it's something new, but it's the same thing [health care providers] have been doing.

Human resource shortages are a major challenge in all 3 countries and affect access to quality of care. Zambia was 28,000 health professionals short of its Ministry of Health staffing targets. In Malawi, interviewed stakeholders said many providers were over-diagnosing malaria because of high client loads (implying not enough trained staff), lack of time or skills for proper clinical diagnosis, and the propensity for providers and clients alike to presume all fevers are malaria.

### Community Engagement

All 3 countries were actively supporting community involvement to enhance and engender community education and mobilization. In Malawi, community groups (for example, village health committees and health surveillance assistants) actively partnered with health facilities to raise awareness in communities about health issues, including MIP; to refer women to ANC; and to identify key health problems in the community.

In Zambia, community groups (for example, neighborhood health committees and safe motherhood action groups) were trained to sensitize communities about a range of maternal health topics in order to promote healthy behaviors and improve care seeking, including for MIP. Although one program follow-up report noted increased ANC attendance in the year since the community program was implemented, no improvements were seen in IPTp coverage due to frequent SP stockouts and poor recordkeeping. At the time of data collection, support for community involvement was not consistent, and more strategies were required to adequately involve communities and foster links between communities and facilities in a sustainable way.

Although there is little documentation and assessment of these community-focused efforts, qualitative feedback suggests they were effective. For example, in Senegal, stakeholders attributed improved ITN use among pregnant women to the USAID-supported “Community Action against Malaria and Tuberculosis” program and in Malawi, to community involvement.

Qualitative feedback suggests that community involvement helped to improve use of bed nets.

### Monitoring and Evaluation

MIP indicators are generally tracked through a variety of sources in malaria-endemic countries, including: (1) periodic household surveys, such as Demographic and Health Surveys, Multiple Indicator Cluster Surveys, and Malaria Indicator Surveys; (2) the routine national HMIS; and (3) sentinel site surveillance systems (now less common). In the 3 countries, health facilities routinely collected and reported data on IPTp uptake by dose during ANC services through the HMIS, which aggregates data into national coverage indicators. However, the WHO-recommended MIP indicators on “percentage of ANC staff trained in the control of MIP in the past 12 months” and “percentage of screened pregnant women with severe anemia in the third trimester, by gravidity”^22^ were notably missing from national data sources in all 3 countries, although data on anemia are recorded in some form at the facility level, most frequently in client records.

ITN indicators have been effectively integrated into the HMIS in Senegal but were absent from the HMIS in Malawi and Zambia. Instead, such information was collected on parallel, program-specific reporting forms. HMIS data quality in Malawi and Zambia was reported to be weak by key informants, but Zambia was making strides to train district medical offices and health facility staff in data management. Prior to data collection, Senegal had taken significant steps, including hiring of new staff and establishment of a web-based data management system, to improve data completeness, timeliness, and reliability.

While SP stockouts were reported to be a significant inhibiting factor for MIP programs, data on stock levels were not routinely made available to reproductive health and malaria control stakeholders. Additionally, at the time of data collection, no countries were reporting national data on MIP incidence or treatment—crucial indicators for measuring the effectiveness of MIP interventions.

### Financing

All 3 national governments commit some funds to MIP programming but still rely heavily on donor support. The Senegalese and Zambian governments were providing funding for SP, with central-level shortages reportedly due more to poor quantification of necessary medicines than to lack of funding. For ITNs, the 3 countries relied entirely on donors, primarily the Global Fund and PMI, which has proved insufficient to cover all pregnant women. While the “Sector-Wide Approach”—a resource allocation system that pools all health sector resources from all sources and allocates funds based on need—had been effective in Malawi for coordinating donor support, lack of accountability of funds provided directly to the government limited contributions to the basket fund. Delays in receipt of Global Fund monies in Malawi and Zambia further hindered program implementation. Across sub-Saharan African countries, funding to support malaria programming has increased since 2004; however, the currently available funding is far below the estimated US$5.1 billion required each year to reach universal coverage of interventions. In 2012, the global total of international and domestic funding for malaria was US$2.5 billion—less than half of what is needed.^27^

## DISCUSSION

Although each country's MIP programming experiences, situation, and needs are unique, Malawi, Senegal, and Zambia all had issues in the areas of commodities, quality assurance, and financing that, with attention, could contribute to improved coverage and MIP outcomes. Careful consideration should be given to addressing all health system areas—even areas that rank high—since the elements are interconnected, and weaknesses in one area can negatively impact other areas. This is especially true in the area of integration, which includes the critical partnership between national reproductive health and malaria control programs to coordinate, plan, and harmonize policies and support synchronized implementation.

MIP programs should consider all areas of the health system to improve MIP outcomes.

Even though the 3 countries studied were considered high-performing MIP countries, none had achieved full coverage of IPTp uptake and ITN use among pregnant women. This is not surprising given each country's low scores in the crucial aspects of financing and commodities. The case studies highlight important lessons learned in each of the 8 MIP program areas that other countries can apply to improve their programs.

In the area of **integration**, lessons focus on strengthening or creating national technical working groups that include the NRHP, NMCP, and National AIDS Control Programme (NACP) to ensure harmonized policies, guidelines, and educational materials, as well as guarantee effective and coordinated (non-duplicative) implementation. A focal point person responsible for coordinating MIP programs across sectors can help ensure consistency and maintain political will.

A focal point person for MIP programming can help maintain political will.

For the **policy** component, the case studies demonstrated that MIP policy should not only be in line with WHO-recommended guidelines but also be interpreted and presented in a consistent manner across all MIP-relevant documents. The October 2012 updated WHO policy recommendation for IPTp-SP—recommending IPTp begin as early as possible in the second trimester and be given at each routine ANC visit at least 1 month apart thereafter—affords countries an excellent opportunity to reinvigorate their MIP programs and review and revise policies and service delivery guidelines. If led by the NRHP and properly coordinated with the NMCP, countries could attract the support—technical and financial—needed to harmonize and disseminate updated guidelines and ensure consistent delivery of MIP services.

Lessons about **commodities** include the value of well-circulated guidance and consistent supervision to ensure that SP is reserved only for pregnant women, thereby ensuring adequate commodities for IPTp and curtailing resistance due to SP misuse. Additionally, coordination of supply chain management and skills in quantification and distribution must be improved. Without consistent supplies of MIP commodities, all other program efforts to increase coverage of interventions will be rendered ineffective.

**Quality assurance** mechanisms that target onsite managers and providers with quality improvement tools that allow for self-assessment can alleviate the barrier of financial and human resource constraints. Self-assessment can further encourage health care providers to find local solutions to address performance gaps.

**Capacity building** was most successful when it focused on both preservice education and in-service training so that health care providers enter the workforce with up-to-date knowledge and skills, and costly in-service trainings can be reserved for periodic updates on MIP guidelines. Strengthened preservice training and alternative in-service capacity-building strategies, such as on-the-job training and mentorship, could contribute to improved training but currently lack emphasis in national programs.

**Community involvement/engagement** was a highly visible component of each country's program. While the programs focused primarily on traditional health messaging and communication to promote ANC attendance and use of ITNs and IPTp, there was also some evidence of efforts to link the community with facility-level care to support better delivery of commodities (for example, delivery of ITNs to communities through health facilities) and access to care. For community-level interventions to be successful and sustained, the communities they serve need to take ownership of their activities and responsibility for supporting them.

In terms of **monitoring and evaluation** processes, MIP indicators related to IPTp and ITNs are being collected through routine and periodic mechanisms but not necessarily captured and aggregated through the HMIS. Since tracking of ITN provision during ANC was not integrated into the HMIS in 2 of the 3 countries, this created challenges for national synthesis and reporting. In addition, information on case management of MIP was lacking in all countries.

To improve MIP monitoring, including data quality and use, priority indicators related to WHO's 3 approaches to MIP prevention and control must be integrated into the national HMIS. District officers and providers must also be trained on data collection, aggregation, and use of data for decision-making, and routine monitoring support must be incorporated into supervision and quality assurance efforts.

Priority indicators must be integrated into the national health management information system.

Finally, **financing**, although a problematic area, was not without solutions. Lessons learned included the importance of advocacy to build in-country awareness from the community to the national level for more dedicated support to MIP programming. By gradually increasing national budgetary inputs to MIP interventions, countries can combat donor fatigue and improve program sustainability.

The Expanded MIP Table of Analysis used to synthesize the 3 country case studies can be adapted to the needs of other countries interested in assessing the status of their own MIP programs. Similar case studies have been conducted in Burkina Faso, India (Jharkhand State), and Rwanda to comprehensively review all technical aspects of their malaria program. This simple and straightforward matrix is a promising tool to help countries move their MIP programming forward and accelerate progress toward national scale up.

The findings from the case studies have been disseminated through workshops in Malawi, Senegal, and Zambia. Following the dissemination workshop in Senegal, country stakeholders began using the recommendations to inform development of its 2012 Roll Back Malaria roadmap. Specifically, Senegal planned to accelerate free distribution of ITNs during ANC and to integrate reproductive health and malaria programs by developing a joint coordination committee, with a special focus on training and community-level interventions. In Zambia, a FANC technical working group was formed, with representation from the RHU, NMCP, and other relevant partners. This group is finalizing and moving forward an action plan of MIP program priorities, including introduction of the new WHO policy for IPTp.^8^

### Limitations

The country case studies were limited to secondary data gathered through the desk review process, as well as qualitative data collected through in-depth interviews among key stakeholders at the national level. Due to time and resource constraints, the country case studies did not generally include interviews with key stakeholders at the regional, district, or facility levels. In Malawi, one District Medical Director was interviewed. Hence, the case studies present a limited but comprehensive picture of MIP prevention and control programming at the national level. Also, since the case studies only targeted better-performing countries, it is not possible to compare the findings to lower-performing countries that could reveal new insights to existing challenges in MIP programming.

Further, while the review is meant to focus on MIP comprehensively, the focus is primarily on IPTp uptake and ITN use and does not address case management in detail, because very little information about case management of pregnant women was found at the country level.

## CONCLUSIONS

This review is particularly important now following the release of the new WHO IPTp guidance as countries review and update national MIP policies. The timing affords countries the opportunity to reprioritize MIP programming and reinvigorate partnerships between reproductive health and malaria control programs, to ensure effective technical oversight and program management. As countries move forward with MIP program acceleration and scale up, it will be important to keep in mind the 3-pronged approach to MIP control, comprising IPTp uptake, ITN use, and case management. Addressing the 3 approaches across each of the interconnected health system areas for MIP lends to a holistic approach to strengthening the health system and lasting results. With this in mind, countries should consider bolstering MIP case management—there is very little data or information to inform what is happening at the country level—as a core component of comprehensive MIP control. As malaria transmission patterns change from high to low, case management of pregnant women will increasingly become more important. The findings from this review as well as the country application of the Expanded MIP Table of Analysis are important tools for countries to review and apply to continue increasing coverage and improving MIP outcomes, and for moving MIP from neglect to priority.
